# Effect of Equipment on the Accuracy of Accelerometer-Based Human Activity Recognition in Extreme Environments

**DOI:** 10.3390/s23031416

**Published:** 2023-01-27

**Authors:** Stephen Ward, Sijung Hu, Massimiliano Zecca

**Affiliations:** Wolfson School of Mechanical, Electrical and Manufacturing Engineering, Loughborough University, Loughborough LE11 3TU, UK

**Keywords:** accelerometer, inertial measurement unit, human activity recognition, wearables, machine learning, extreme environments

## Abstract

A little explored area of human activity recognition (HAR) is in people operating in relation to extreme environments, e.g., mountaineers. In these contexts, the ability to accurately identify activities, alongside other data streams, has the potential to prevent death and serious negative health events to the operators. This study aimed to address this user group and investigate factors associated with the placement, number, and combination of accelerometer sensors. Eight participants (age = 25.0 ± 7 years) wore 17 accelerometers simultaneously during lab-based simulated mountaineering activities, under a range of equipment and loading conditions. Initially, a selection of machine learning techniques was tested. Secondly, a comprehensive analysis of all possible combinations of the 17 accelerometers was performed to identify the optimum number of sensors, and their respective body locations. Finally, the impact of activity-specific equipment on the classifier accuracy was explored. The results demonstrated that the support vector machine (SVM) provided the most accurate classifications of the five machine learning algorithms tested. It was found that two sensors provided the optimum balance between complexity, performance, and user compliance. Sensors located on the hip and right tibia produced the most accurate classification of the simulated activities (96.29%). A significant effect associated with the use of mountaineering boots and a 12 kg rucksack was established.

## 1. Introduction

In extreme environments, such as remote high mountains, emergency response is often limited, and therefore, identifying negative health trends is a critical issue. There are a vast range of reasons why humans ascend to altitude and expose themselves to this harsh environment. One common purpose includes recreation in the form of mountaineering and trekking. To support these recreational users, multiple professionals are often also required, and consequently there is a huge leisure and tourism industry employing mountain guides and porters/Sherpas. There are also native inhabitants of mountain regions, with over 81 million people estimated to live higher than 2500 m above sea level [1]. Mountains are inherently dangerous places, with extreme environmental conditions such as high wind speeds, low barometric pressure, and low temperatures. Alongside famous incidents, such as the 1996 Mount Everest expedition where eight climbers died when a storm trapped them on the mountain, numerous health events lead to abandoned objectives and medical evacuations. Helicopter medical evacuation rates to a single hospital in Nepal amounted to 905 per 100,000 mountaineers in 2017 [2]. Studies have also shown incidence rates of acute mountain sickness, (a common form of illness associated with high altitude) exceeding 40% when ascending above 2500 m [3]. This condition alone therefore has the potential to affect millions of people annually.

To mitigate the risk posed to these groups their health must be monitored. In order to truly understand the health of an individual, first the activities of the individual must be fully identified. This preliminary study is part of a wider initiative aimed at developing an innovative solution to health monitoring in extreme environments.

## 2. Related Work

Physical activity in the presented mountaineering context can be better described as movement between static postures such as sitting or standing, or a dynamic motion such as walking or climbing [4]. The need to quantify and better understand physical activity is applicable to a wide range of fields, including healthcare, physical training, and sports [5,6,7].

Multiple methods exist to quantify physical activity, ranging from simple approaches through to fully automated artificial intelligence methodologies. Each method has its own relative strengths and weaknesses. Likewise, the nature of the environment within which the activity monitoring takes place will also have a substantial influence on the selection of an effective method. The existing literature has demonstrated a clear need to understand more than just a binary state of either active or sedentary behaviour, and in light of this, the field of activity recognition and human activity recognition (HAR) has been established [8]. A little explored application of HAR is that of people operating in extreme environments, and the respective need to understand what activities they are completing. Whilst traditional monitoring methods such as simple observation have been shown to be effective in controlled environments, such as during sports training sessions, it is not possible to have observers following participants in dynamic hazardous environments; as such, there is a need for automated monitoring systems.

The two most popular automated activity recognition systems can be classified as either vision-based, or sensor-based. Vision-based systems require the use of cameras and direct line of sight to record data, whereas sensor-based systems require sensors to be affixed to the user. Vision-based systems are not suitable for deployment in extreme environments due to their inherent limitations. With the ongoing development in sensor miniaturisation and the associated reduction in power consumption, wearable sensors have gained popularity as a possible solution within these environments [9].

A frequently utilised wearable sensor is an accelerometer, which measure acceleration. Accelerometers have been demonstrated to be effective at identifying a wide range of human activities [10]. Accelerometers are often included in a range of systems reported in the literature [11], and are used in the identification of physical activity, energy estimation [12], and fall identification [13]. Accelerometers are well suited to extreme environments due to their relatively small size, in addition to being battery powered and operating wirelessly.

The need to optimise the accuracy and performance of HAR systems is well established. A large body of the work within the sensor-based HAR field focuses on feature extraction [14,15] and the development and implementation of classification models [16] to improve recognition accuracy. Whilst these aspects are unarguably essential to improve the performance of HAR systems, they often overlook some of the elements which are most influential in real-world applications.

Notably, one of the more significant challenges is the question of how to improve recognition accuracy with the optimisation of the number and location of sensor nodes [17]. This has been widely studied in relation to activities of daily living [18,19], but has not been explored in relation to activities within an extreme environment. Practical limitations exist on the number of sensors a user will tolerate [20], and the effect they have on the ability of the user to complete their task unencumbered. A compromise must therefore be made between the level of information the system requires and any detrimental effect on the user. Further consideration must also be placed on the errors attributable to incorrect positioning, environmental conditions, and sensor variability [21]. These errors can either be amplified or diminished by different system configurations. Therefore, there is an immediate need to establish if the optimal number of sensors, and their respective configurations, as established in research related to normative environments remains the optimal configurations when deployed into an extreme environment.

A second factor which plays a pivotal role in HAR performance is the training data used in the development of deployable models. It is well understood that activity specific-training data are required to provide accurate activity detection in a supervised machine learning system. Training a system with data pertaining to activities of daily living and expecting it to perform well at detecting specific sporting activities is not feasible. Therefore, systems are trained with representative data of the activities intended to be classified. However, these training data often do not consider the clothing or equipment which will be utilised by the end user. The influence this may have on the overall classifier accuracy is not well established and the nuances of individual use cases such as mountaineering are unexplored. This gap in the existing knowledge base prevents the predictable deployment of current models into extreme environments.

Mountaineers are required to use specialised equipment to protect them from climatic conditions present at high altitude as well as items which aid movement in these environments. Mountaineering participants are required to always carry resources and equipment with them whilst conducting the activities, this is often accomplished using a rucksack containing their provisions. Further, mountaineering boots which possess a very stiff stoles and accommodate a metal crampon are required to provide traction whilst on snow and ice. The effect this equipment will have on classifier accuracy is unknown and requires further analysis to understand the implications which this may have on HAR systems.

It is important to consider the context of the proposed HAR application when proposing an effective system. Once a negative health event has been detected, a response is required. Multiple challenges exist surrounding communication in mountaineering environments. Traditional monitoring systems rely on mobile phone network communication between the user and central command, whereby information and data are passed and a decision is made at the command structure [22]. Mobile phone technology (3G, 4G, 5G) and other conventional communication modalities are often unsuitable for use in extreme environments due to a lack of infrastructure and environmental limitations [23], therefore complex satellite communication systems are often the only effective option. These systems can be expensive and difficult to integrate with monitoring platforms. A novel system was proposed by Galli et al. [24] where they demonstrated the feasibility of a satellite Internet of things (IOT) approach to send monitoring data from an individual to a command structure. Due to the unreliability of two-way communication, a design approach where all processing was done at the edge-device level and contextualised information was passed back to the control structure was established. As such, when considering the optimisation of HAR for mountaineers, resource usage and computational demands must be minimised wherever possible to allow for deployment on limited edge devices.

The purpose of this study was to answer: (I) What is the most accurate classifier for mountaineering specific activities? (II) What is the optimal sensor combination and number, and where should the accelerometers be positioned? (III) What effect does activity-specific equipment have on classifier accuracy?

This pioneering work is the first time HAR has been explored specifically for mountaineers, as well as utilising more extensive sensor locations and configurations to provide a comprehensive evaluation. Further, user-worn equipment is included as a parameter in the analysis of HAR classifiers for the first time. In the following manuscript and coming results, a greater understanding is gained, filling in the gaps between the theoretical knowledge of HAR approaches developed within a laboratory environment and the real-world application of these approaches to a specific user group with unique considerations and requirements. The results obtained allow for the predictable deployment of an effective HAR system into the mountain environment, whereby further optimisation can occur. Additionally, the findings have far-reaching implications beyond the niche of the mountaineering case study presented here.

## 3. Materials and Methods

### 3.1. Participants and Ethics

Eight subjects were recruited to participate in this study, comprising of seven male subjects and one female subject. Subject demographics are summarised in Table 1. Subjects ranged in age from 19–32 years, with a mean age of 25 years. All subjects were students at Loughborough University with previous mountaineering experience. Prior to participating in the trial, all subjects completed a medical screening questionnaire to ensure their suitability to partake in the testing. The study was approved by Loughborough University Ethics Approvals (Human Participants) Sub-Committee (R19-P175).

#### 3.1.1. Instrumentation and Equipment

**Figure 1:**
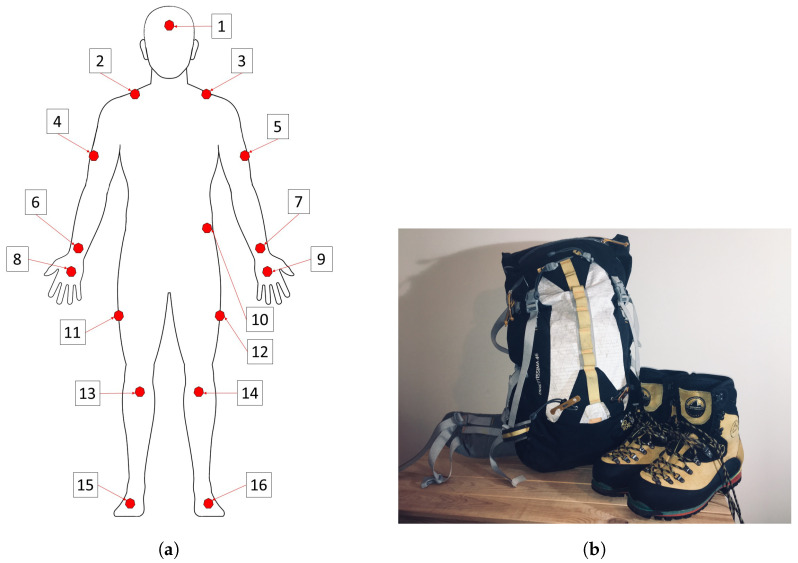
(**a**) Approximation of accelerometer placements from the Perception Neuron inertial motion capture (Mo-Cap) suit (Node 17: C7 vertebrae not shown). (**b**) Example of mountaineering rucksack (Mountain Hardwear, Direttissima 46) and B3 rated boots (LaSportiva, Nepal Evo GTX).

The Perception Neuron inertial motion capture (Mo-Cap) (Noitom International Inc., Miami, FL, USA) was selected for use as it allowed for 17 simultaneous accelerometers to be positioned at various anatomical positions around the body. The suit was configured in the 18-neuron configuration, and was worn by all subjects in the trial. In the 18-neuron configuration, 17 inertial measurement units (IMUs), measuring 12.5 mm × 13.1 mm × 4.3 mm, and containing a 3-axis accelerometer (±16 g), a 3-axis gyroscope (±2000 dps), and a 3-axis magnetometer per unit, are utilised. Only the accelerometer data stream was used in this trial, with the body positions of the sensor locations shown in Figure 1a and detailed in Table 2. The sensors form an integrated part of the proprietary suit and, therefore, the suit was fitted in accordance with the manufacturer’s instructions. Data were sampled at a frequency of 120 Hz. This frequency was chosen as it covered most of the established everyday activity range of approximately 20 Hz, whilst allowing for unknown ranges in the mountaineering specific activities. Additionally, ample bandwidth was captured which allowed us to downsample postcapture if required. Similarly, the accelerometer range was deemed suitable to capture the established normal range of bodily acceleration amplitudes of ±12 g [18]. Data were aggregated in the suits hub and transmitted wirelessly to a PC running the suits’ proprietary software application (Axis Neuron, Noitom International Inc., Miami, FL, USA). The data were then exported from the proprietary software application and imported into MATLAB (Matlab, MathWorks, Natick, MA, USA) for analysis.

#### 3.1.2. Experimental Protocol

The subjects were asked to complete seven whole-body activities which included walking on flat ground, walking up an incline slope, walking down a decline slope, walking up stairs, walking down stairs, sit on a 30 cm high block from standing, and stand from sitting on a 30 cm high block. The trial consisted of two parts corresponding to equipment states, defined as either “unequipped” or “equipped”. Initially, the participants completed each activity whilst wearing standard gym clothes (t-shirt, shorts, and trainers); this equipment state was designated “unequipped”. Next, the participants completed the same 7 activities whilst wearing mountaineering boots (B2 or B3 rating) and a 12 kg mountaineering rucksack. This was designated as the “equipped” state. The same rucksack was used for each trial, with the participants permitted to adjust the straps on the rucksack to their preference. Each participant completed each activity for three discrete repetitions for each equipment state. Data were then manually labelled postcapture by a human observer. Example equipment is shown in Figure 1b.

### 3.2. Feature Extraction

The raw acceleration data were collected for each activity and labelled accordingly. The acceleration signals comprised both a body acceleration component and a gravitational acceleration component. As utilised in previous studies, a fourth-order Butterworth high-pass filter with a cut-off frequency of 0.25 Hz was used to remove the gravitational component and isolate the body acceleration component of the acceleration signal [25,26,27]. In addition to the three orthogonal axes, the three axes were combined to produce the signal magnitude vector (SMV) (Equation (1)).

(1) $$SMV=\sqrt{a_{x}+a_{y}+a_{z}},$$

The three individual axis and SMV acceleration signals were partitioned into 1 s (120 samples), 50 percent overlapping windows. A 50 percent overlap was chosen as it has been shown to produce effective results in previous studies [28,29]. Table 3 presents the number of instances per class. In total, 2701 windows were represented across the 7 activities, and 2 equipment states were investigated.

Multiple descriptive features were then extracted from the collated windowed dataset; an overview of the extracted features is shown in Table 4.

The features as summarised in Table 4 were calculated for all three axes and the combined SMV. The equations for calculating features 33–41 are shown in Table 5:

### 3.3. Classification Models

The application of classification models to solve activity recognition problems is a maturing field, with no universally accepted optimal algorithm for the detection of physical activities. Additionally, there is no precedent within the literature specifically relating to mountaineers and their specific use case. Each classification algorithm has its own relative advantages and disadvantages more thoroughly explored by Nweke et al. [30] and Lima et al. [16]. As such, 5 commonly used machine learning algorithms were selected and evaluated for accuracy. The algorithms evaluated comprised ensemble bagged trees (EBT), support vector machine (SVM), decision trees (DT), weighted *k*-nearest neighbours (*k*-NN1), and *k*-nearest neighbours (*k*-NN2). The configurations of the classification models are shown in Table 6. Each classification algorithm was assessed using a 10-fold cross-validation with 10 iterations for all single, two-, and three-sensor combinations using MATLAB R2020a Statistics and Machine Learning Toolbox Ver. 11.7 (Matlab, MathWorks, Natick, MA, USA). Further analyses of sensor combinations and locations were completed using a 25% holdout cross-validation methodology.

## 4. Results

### 4.1. Classification Model Performance

**Figure 2:**
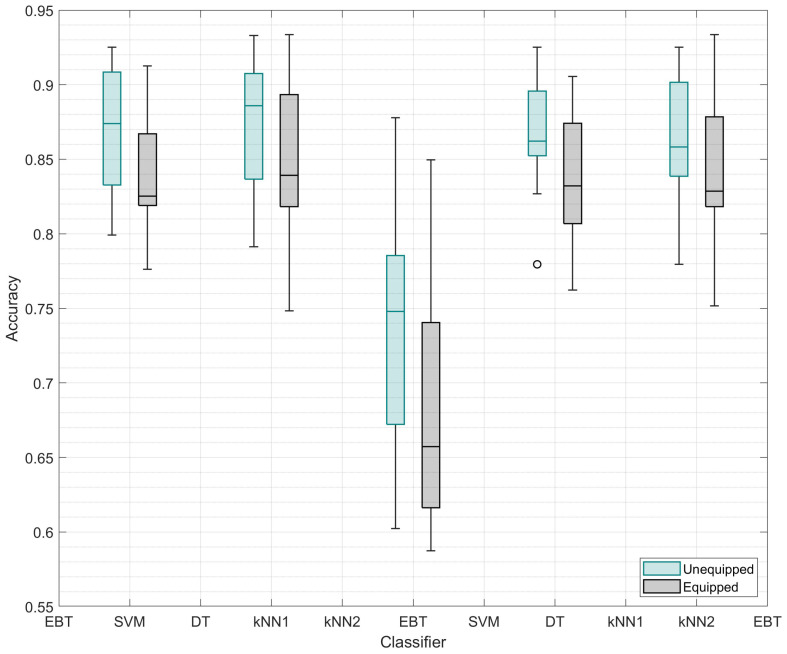
Box plot presenting the classifier accuracy of all 7 activities, for each equipment state, as calculated by each machine learning algorithm for all sensor locations (17 sensor nodes). Note the vertical axis does not start from zero to aid readability.

Single-sensor performance was considered in this initial analysis to determine which classifier provided the most accurate activity detection. Data from both equipment states were evaluated: unequipped and equipped. Figure 2 and Table 7 present the average percentage of correctly classified instances from all sensor locations.

As shown in Table 7, the SVM achieved the highest accuracy for both equipment states in the single-sensor configuration, unequipped (87.51%) and equipped (85.23%). There was a significantly different individual classifier accuracy for the single-sensor configuration in the unequipped state (F(4,64)=66.803,p<0.001). Post hoc testing revealed that the decision tree was the only significant result, performing worse than all other classifiers at 73.06% (p<0.001). Again, there was a significantly different individual classifier accuracy for the equipped state (F(1.806,28.888)=108.785,p<0.001) (Greenhouse–Gessier corrected). The DT was the only significant result (68.61%,p<0.001), where the DT classifier achieved significantly less accuracy than the SVM (84.78%). EBT (83.97%, p=1.00), *k*-NN1 (83.97%, p=1.00), and *k*-NN2 (84.28%, p=100) all performed worse than the SVM but were not statistically significant.

### 4.2. Effect of Equipment

As shown in Table 8, similar results were observed for natively trained data sets where the equipment loading status remained constant throughout the training and evaluation phases. The mean classifier accuracy values were 83.36% (unequipped) and 81.15% (equipped). When the model was trained with data from the unequipped state and then tested with data from the equipped state, the classifier accuracy fell significantly across all classifiers (mean classifier accuracy of 51.79%).

### 4.3. Sensor Combinations

**Figure 3:**
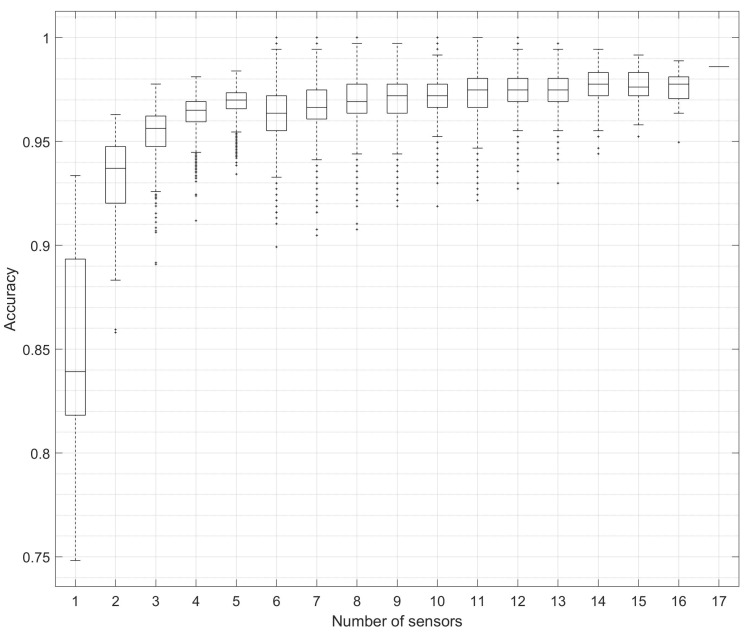
Box plot presenting the SVM classifier accuracy for each sensor configuration, single to 17-sensor combinations. Note the vertical axis does not start from zero to aid readability.

All possible sensor combinations (131,071) were analysed using the equipped state dataset and the SVM classifier; results are shown in Figure 3 and Table 9.

A Kruskal–Wallis H test was used to establish if there were differences in activity classifier accuracy for different combinations of sensor locations, ranging from a single sensor to 17-sensor combinations. Dissimilar distributions of classifier accuracy were noted via visual inspection of a box plot. It was established that the distributions of classifier accuracy were significantly different between groups, $X^{2}\left(16\right)=11,140.864,p<0.001$.

Post hoc testing comprised of pairwise comparisons using Dunn’s procedure, with a Bonferroni adjustment for multiple comparisons, was performed. Adjusted *p*-values are presented, and the displayed values are the mean ranks. There was no significant difference between one sensor (56.56) and two sensors (3449.54) (p>0.05), one sensor and three sensors (20, 528.07) (p>0.05), and one sensor and 17 sensors (125,337.50) (p>0.5). All remaining comparisons of one sensor, and four to sixteen sensors, produced significant differences (p<0.005).

### 4.4. Optimal Sensor Location

Table 10 shows the ten highest and the lowest ranked sensors or sensor combinations for the equipped state dataset, using the SVM classifier.

## 5. Discussion

### 5.1. Classifier Performance Analysis

Extensive literature exists on the general optimisation of activity recognition classifiers, such as model tuning [31], and feature selection [10], so this was not the main focus of this paper. Rather, attention was placed on the less-well-explored factors which can affect overall classifier performance. As far as the authors are aware, there is no literature surrounding the selection of classification models for a mountaineering setting. Therefore, an evaluation of previously used classifiers was completed on the single-sensor configuration, to determine which classifier performed best at identifying mountaineering-specific activity.

The results demonstrated that the SVM achieved the highest accuracy over the seven tested activities. Due to this highest overall accuracy, the SVM was chosen for the further analysis of other factors associated with activity recognition conducted within this study. Given that the other classifiers achieved similar results, it could be reasonably argued that they could have been used in place of the SVM, as the small difference noted would likely not have a significant impact in real-world applications. The only classifier which performed with consistently significantly lower accuracy was the decision tree, and as such, it would not be recommended to use this classifier in this application.

We selected existing signal processing and classification techniques which are well-known and well-understood within the scientific literature to remove the uncertainty which could be associated with a new method. The chosen protocol approach of utilising a reliable method made it possible to perform a more concise analysis of the unique study aims, contributing to the existing state-of-the-art research. The following sections each provide a previously unexplored insight into HAR systems for mountaineers.

### 5.2. Number of Sensors

The optimal number of sensors has been explored previously, with studies examining the effect of multiple sensors for everyday activities [18,32]. However, this is the first time that data from 17 individual sensors have been captured simultaneously, a far greater number than in previous studies. Whilst the implications of using such a large sensor set would make the system impractical for real world deployment, it allows a greater level of analysis to be conducted on subsets of sensors, from which, optimised deployable systems can be devised. Moreover, this study is the first to consider the effects of the equipment required by the intended end user. Therefore, the results gained are more applicable to the deployment of systems into extreme environments than the existing literature relating to HAR for everyday activities.

The results from the study showed that there was a large increase (+8.49%) in mean classifier accuracy from one sensor (84.78%) to two sensors (93.27%), followed by only small increases for each additional sensor, (3 sensors (+2.1%), 4 sensors (+1.0%) and 5 sensors (+0.56%), with a reduction then noted with 6, 7, and 8 sensors, and then a further small incremental increase with each sensor addition up to maximum percentage accuracy at 17 sensors (98.60%). These results suggest that the classifier accuracy is improved through the addition of more sensors. However, classifier performance alone is not the only absolute determining factor when designing a wearable system. With the addition of more sensors, there is added complexity and a greater requirement for computational resources. For laboratory-based settings, the use of a high-powered PC poses little difficulty. However, when deployed on an edge device in an extreme environment, additional factors such as limited computational power and a reduction of battery efficiency in cold weather must also be considered. Therefore, computational resource requirement must be minimised. As such, further work is needed to optimise the system and reduce the feature set as much as possible without losing accuracy.

To gain optimal compliance with wearable systems, the burden on the wearer must be reduced wherever possible. If separate devices are to be used, they must be individually managed, including power and charging, synchronisation, and physical attachment of the sensor to the person. In a laboratory-based research study, this is little more than an added complexity that can be easily overcome. Yet, when applied to an extreme environment, these issues can become a lot more problematic.

For use in an extreme environment, it is prudent to keep a system as simple as possible and thereby reduce the possible failure modes. From the results gained, it was shown that a mean target classifier accuracy of >95% was achievable with three sensors (95.37%). However, this target can also be achieved by two sensors where the maximum accuracy was shown to be 96.29%. This was achieved when the selection of a sensor pair and their respective locations were optimised. It was, therefore, deemed that there were diminishing gains when more than two sensors were used and the balance between performance, usability, resource cost, and complexity, became unfavourable.

### 5.3. Location of Sensors

As shown in Table 10, the location of the accelerometers can have a direct influence on the classifier accuracy. When single sensor locations were ranked in isolation, the feet provided the highest classifier performance across the full range of activities, followed by the lower legs, hips, and spine. The classification accuracy was below 85% for all remaining sensors.

However, different activities involve the motion of different body segments and, as such, the positioning of the sensor can directly influence performance. For example, during the stand-to-sit activity, very little motion will be noted in the foot and, therefore, the predictive accuracy of the classifier to differentiate if the person is standing or sitting based on this data stream is low. Alternatively, during walking, the amount of movement in the foot is high and, therefore, is more likely to produce a higher classifier accuracy.

The situation becomes more complex when trying to differentiate between similar activities, such as walking on flat ground and walking up an incline. In general, the sensors attached to the lower legs and feet (89.16–93.36%) provided the best data for activity recognition over the seven activities, with the hands and lower arms (74.83–81.82%) performing the worst. This could be attributed to the nature of the activities performed, and the fact that they did not require consistent use of the upper body to complete the activity. Rather, the upper body was more susceptible to individual variations not directly related to the task, such as the amount of swing in the arms whilst walking, or the use of the arms as support during the transition from standing to seated or vice versa. Via observation, it was noted that not all participants used their hands to guide themselves when transitioning between sitting and standing states. For the participants which did utilise their upper body for assistance, there was a large variation in the use of a single hand, or two hands, and the amount of support required. As a result, in complex activities such as these, rather than relying on a single sensor, multiple sensors can be combined to give a deeper understanding of the movement, thus improving classification accuracy. Therefore, all possible combinations of two sensors (136 permutations) and single sensors (17 permutations) were considered in this stage of the analysis. The highest accuracy with two sensors was achieved with the hip and right tibia sensors (96.29%). A further 24 pairs achieved a greater than 95% accuracy, the top 10 pairs are shown in Table 10.

Overall classifier performance is not the only factor requiring consideration when choosing a sensor location; rather, the usability and environmental constraints must also be considered. For this particular use case the ability to withstand extremely cold temperatures, exposure to water, and impact resistance must all be considered.

### 5.4. Equipment State

A widely overlooked aspect of activity recognition is the effect application specific equipment has on the overall classification accuracy. Previous studies often looked to validate a classifier for a specific purpose, with little attention paid to extrinsic elements which could change the results of the classifier. It is not known what effect a change in equipment, such as clothing or additional loading, would have on the classifier. A key contribution of this study was to directly address this uncertainty.

Similar results were gained from both training the classifiers without equipment (mean classifier accuracy 83.36%) and with equipment (mean classifier accuracy 81.15%). Results were consistent over all five classifiers for one sensor, two-, and three-sensor combinations. Generally, classifier accuracy was approximately 1% higher for the unequipped state over the equipped state. The decision tree was an outlier with a larger approximate 4% difference being noted. The largest differences occurred when the classifier was trained without equipment, and then the same activity protocol was completed in the same environment, with only a change of footwear and the addition of a 12 kg rucksack, with the classifier then used to test this dataset. Results were approximately 30% worse on average, with individual sensor locations achieving as low as 30.13% accuracy.

This large variation in classifier accuracy presents significant challenges when designing an activity recognition system for deployment in hostile and extreme environments, especially where additional clothing in the form of personal protective equipment (PPE) and additional loading (weight of equipment, rucksack, etc.) are common. To improve the system performance, variations in task completion due to these additional loadings must first be understood.

Previous studies have shown that changes in posture such as trunk angle, gait pattern, and stride length [33,34] are observed when carrying additional loads. This is caused by the larger forces which the body must generate through the muscles to propel and control a larger mass [35]. Further compounding these changes, the second additional loading element explored within this study was the use of mountaineering boots. Typically, mountain boots are heavier than everyday footwear, and are notably restrictive of the ankle joint, effectively immobilising it. All these factors work together to influence the gait cycle of the user. Previous studies have shown that single leg support times increase, and double leg support times decrease, with the use of mountaineering boots [31]. This change in gait cycle causes resultant changes in the acceleration profile, as seen by the accelerometers and, as such, the classification algorithm pattern recognition leading to a reduced accuracy. Different sensor locations have varying susceptibility to this change, with more generalised changes noted across all sensors. Additionally, specific sensors may be altered by human factors not related to locomotion, but to participant behaviour. Anecdotally, it is common for people wearing a rucksack for long periods of time to rest their hands upon the rucksack shoulder straps. This change in behaviour cannot be attributed to a specific activity and may not exist in all instances. This demonstrates human variability which poses a particular challenge when trying to automate analysis, and effective inspection of these factors is required to better understand sensor location selection and sensor configurations. By using multiple sensors distributed about the body, the influence of individual body segments on the overall activity classification can be reduced. Within the range of activities explored in this study, the arms played little role in the completion of the activities, and by choosing sensors placed elsewhere, the classifier accuracy could be improved.

Additionally, the use of equipment is a factor which requires consideration when choosing sensor locations for specific applications. The accuracy of the classifier was shown to be significantly worse for the shoulder sensors when equipment was used. The accuracy of the SVM classifier with the left shoulder node as the input was 10.31% less accurate and the right shoulder 6.98% less accurate. This reduction could have been caused by the interference of the rucksack with the fixed mountings of the shoulder sensors on the MOCAP suit. However, the effect of this conflict is expected to be minimal as it was possible to position the sensors in the correct location and orientation after the rucksack was put on. There was also no contact between the rucksack straps and the sensor units present. In other applications, this may not be as easy to overcome, and alternatives may need to be found.

Whilst body-worn locations were evaluated within this study, it is important to recognise the aim of the system is to detect a specific activity. Therefore, the sensor does not need to be exclusively body-worn with skin contact. Activity detection may be able to be achieved through monitoring of an external piece of equipment, for example, rucksack or helmet motion. This potential approach would require further research and is beyond the scope of this paper.

### 5.5. Significance

There are multiple implications when considering the use of classification algorithms where external, activity-specific equipment is likely to be used. In respect of training data, a lack of generalisation has been established. A significant reduction in classifier performance is present when equipment is used with a classifier not trained with it. The use of trained large-scale models deployed on commercial devices, even with representative activities, will not achieve adequate performance if additional equipment or loading is utilised. It has, therefore, been established that all models need to be trained with data using the same equipment that will be used in the intended setting. As far as the authors are aware, this is the first study to explore the specific effect of specialised equipment and loading conditions on activity recognition classifier accuracy during simulated mountaineering activities.

The unique findings presented here relating to the effect of equipment and loading are applicable to a large range of fields beyond the case study of mountaineers. They will be equally applicable to other users in extreme environments, or any application which requires specific clothing or equipment to be used. This far-reaching user base includes first responders, exposed workers, and military personnel. It also raises doubts around the reliability of activity classification in more general use cases, such as activity measurement in rehabilitation. The use of orthoses, prostheses, and aids such as crutches may have a similar negative effect on activity recognition classifier accuracy. This could have substantial implications on the clinical validity of such devices and metrics gained in these situations. The accuracy of activity recognition embedded within widely used consumer devices associated with sport, recreation, and activities of daily living is also brought into question.

### 5.6. Limitations

When considering these results, a number of limitations must be taken into account. During the design of the study, it was envisaged that a larger number of participants would be recruited into the trial to boost its statistical power. However, due to the COVID-19 pandemic, this was not possible, and in line with these constraints, the number of participants was reduced to eight. All participants were relatively young and healthy with good levels of general fitness. This small sample size reduces the confidence in generalising the results found to the wider population, especially older, less healthy individuals. Therefore, future work should aim to recruit a larger number of participants, and a more balanced gender distribution.

Due to the nature of the proprietary Mo-Cap suit, there were inherent limitations to the sensor placements. Namely, the intertwined structure of the sensors distributed about the suit meant the sensors were restricted to the locations the manufacturer selected during the suit’s design. As all the sensors were affixed to the suit, movement could cause the elastic fabric to be pulled and create movement artefacts elicited at one or more sensors. Further experiments should aim to use independent sensors which are not affected by the movement of other items of clothing or equipment.

The intention of the paper was to draw attention to the widely overlooked aspects of human activity detection, most specifically the use of activity-specific equipment and its effect on activity classification. As such, a relatively simple identification method was adopted to illustrate these effects without the unnecessary complication of more complex approaches. Indeed, for future research and deployment into extreme environments, further work is required in optimising the classification methods to improve real-world classification performance. The paper presented contributed to the theoretical foundation required for this future work to be completed.

The classification algorithms within this study utilised only accelerometer data. However, it has been shown that a greater accuracy can be gained by using sensor fusion approaches, including common sensors such as gyroscopes and magnetometers. Despite these data streams being collated during testing, they were not utilised in the analysis. The aim in this application was to reduce complexity wherever possible, due to the challenges presented in extreme environments. By using a single sensor, power usage is reduced at the sensor level, and a reduction in computational resources at the processing-node level is achieved. Magnetometers are vulnerable to magnetic interference, which is often present due to certain magnetic rock formations existing in mountainous environments. Similarly, the cold temperatures often experienced in mountainous regions can cause a drift in gyroscopes which requires calibration and compensation.

Lastly, the data used within this study were captured under controlled laboratory conditions, with direct supervision of the activities by the researchers. Factors outside of the laboratory, such as uneven ground and the traction effects of slippery surfaces such as gravel and snow also require consideration. Therefore, the efficacy of using simulated equipped state data from laboratory settings in real-world applications is yet to be determined and requires further testing for validation.

## 6. Conclusions

Accelerometers have been widely used in the field of activity detection within normative environments. However, less attention has been paid to complex applications such as mountaineers operating within extreme environments. In these contexts, the ability to accurately identify activities has the potential to prevent death and serious negative health events to the operators. This study aimed to be the first to investigate factors associated with the application of human activity recognition to simulated mountaineering activities, whilst wearing appropriate equipment, thus filling the gaps between theoretical HAR approaches developed within a laboratory environment and the real-world application with user-group-specific considerations, limitations, and requirements.

The first aim of the study was to select which machine learning classifier performed the best at classifying mountaineering-related activities. The accuracy of activity classification based on data recorded simultaneously from 17 body worn accelerometers was tested. The results demonstrated that the SVM provided the most accurate classifications of the five machine learning algorithms tested. The EBT, *k*NN1, and *k*NN2 performed marginally worse, and the DT produced significantly poor results.

The second aim of the study was to identify the optimum number of sensors and their respective body locations to achieve the most accurate activity detection. A comprehensive analysis of all possible combinations of the 17 accelerometers was performed. It was found that two sensors provided the best balance between complexity, performance, and user compliance. The inclusion of additional sensors only achieved marginal improvements with impractical implications. Sensors located on the hip and right tibia produced the most accurate classification of the tested simulated mountaineering activities. Data could also be used from the hips, paired with the left tibia, with negligible difference.

Finally, the third aim of the study was to explore the effect that activity-specific equipment had on the classifier accuracy. A significant effect associated with the use of mountaineering boots and a 12 kg rucksack was established and, therefore, the need to train any machine learning classifier with representative equipment being utilised was noted. The use of standard trained models, even if representative of the activities, are unlikely to reach desirable levels of accuracy if additional equipment is being worn by the user. This has implications reaching far beyond the niche of the mountaineering case study presented here, with the potential to effect HAR classifier design and training in any situation where additional equipment or loading is present.

The results gained from the exploration of HAR for mountaineers is worthy of further attention. The development of HAR approaches beyond the traditional methods presented here will be expressed in future work, whereby we will expand on these findings by conducting further testing outside of the sterile laboratory in real-world extreme environments.

## Figures and Tables

**Table 1:** Participant demographics.

	Mean ± SD (n = 8)	Minimum	Maximum
Age (years)	25.0 ± 4.3	19.0	32.0
Body Mass Index (kg m^−2^)	23.7 ± 3.9	17.6	28.6

**Table 2:** Perception Neuron anatomical IMU positions.

Sensor Node	Anatomical Position
1	Occiput
2 and 3	Acromia (L & R)
4 and 5	Centre of humerus (L & R)
6 and 7	Distal ulna (L & R)
8 and 9	Dorsum of hand (L & R)
10	Hips
11 and 12	Midshaft lateral femur (L & R)
13 and 14	Proximal tibia (L & R)
15 and 16	Dorsum of foot (L & R)
17	C7 vertebra

**Table 3:** Number of windows per activity.

Activity	No. of Windows	
	Unequipped State	Equipped State
Walking on flat ground	205	227
Walking up incline	238	248
Walking down incline	253	266
Walking up stairs	226	269
Walking down stairs	214	263
Stand from seated	66	66
Sit from standing	69	91

**Table 4:** Extracted features.

Feature No.	Feature	Feature Description
1–4	Mean value	Mean value of x-, y-, z-, and signal magnitude vector (SMV)-windowed values
5–8	Variance	Variance of x-, y-, z-, and SMV-windowed values
9–12	Standard deviation	Standard deviation of x-, y-, z-, and SMV-windowed values
13–16	Maximum	Maximum value of x-, y-, z-, and SMV-windowed values
17–20	Minimum	Minimum value of x-, y-, z-, and SMV-windowed values
21–24	Mean absolute deviation	Mean absolute deviation of x-, y-, z-, and SMV-windowed values
25–28	Range	Range of the x-, y-, z-, and SMV-windowed values
29–32	Root mean square	Root mean square of x-, y-, z-, and SMV-windowed values
33–36	Skewness	Skewness of x-, y-, z-, and SMV-windowed values
37–40	Kurtosis	Kurtosis of x-, y-, z-, and SMV-windowed values
41	Spectral energy	Energy of SMV-windowed values

**Table 5:** Calculation of extracted features.

Feature	Equation
Skewness	(2) $Ske_{w}=\frac{\frac{1}{N}\sum_{i=1}^{N}{\left(w_{i}-\mu \right)}^{3}}{{\left(\frac{1}{N}\sum_{i=1}^{N}(w_{i}-\mu {)}^{2}\right)}^{3}}$
Kurtosis	(3) $Kur_{w}=\frac{\frac{1}{N}\sum_{i=1}^{N}{\left(w_{i}-\mu \right)}^{4}}{{\left(\frac{1}{N}\sum_{i=1}^{N}{\left(w_{i}-\mu \right)}^{2}\right)}^{2}}$
Spectral energy	(4) $$Energy_{x}=\frac{\sum_{i=1}^{\left|n\right|}{\left|x_{i}\right|}^{2}}{\left|n\right|}$$ $x_{i}$ denotes the Fast Fourier transform (FFT) components of the window.

*w_i_* denotes either the *a_x^-^_*, *a_y^-^_*, *a_z^-^_*, or *SMV*-windowed values, with *N* observations contained within the window.

**Table 6:** Classification model configuration.

Classification Model	Configuration
Support vector machine (SVM)	A cubic kernel function and automatic box constraint and kernel scale defined by Matlab’s inbuilt heuristic procedure which utilises a subsampling methodology was used. A multiclass method which reduced the multiclass configuration into a series of binary classification subproblems using a “one-vs.-one” approach was also utilised.
Ensemble bagged trees (EBT)	Utilised Breimans’s “random forest” algorithm. A maximum number of 1429 splits was established, and the number of learners was set to 30.
Decision tree (DT)	Maximum number of splits set at 100; Gini’s diversity index was used to define the split criterion.
*k*-nearest neighbours (*k*-NN1)	Medium distinctions between classes, using a Euclidean distance weighting. A squared inverse weighting function was implemented where ($weight=\frac{1}{distance^{2}}$). The number of neighbours was set to 10.
*k*-nearest neighbours (*k*-NN2)	Fine distinctions between classes with no Euclidean distance weighting were applied. The number of neighbours was set to 1.

**Table 7:** Percentage of correctly classified instances of all 7 activities for each equipment state as calculated by each machine learning algorithm. Results present the mean classifier accuracy of all sensor locations (17 sensor nodes), classification produced through 10-fold 10-iteration cross-validation. * denotes significant difference in percentage classified.

State	Classifier								
	SVM	EBT		DT		*k*-NN1		*k*-NN2	
	Accuracy (%)	Accuracy (%)	(*p*-Value)	Accuracy (%)	(*p*-Value)	Accuracy (%)	(*p*-Value)	Accuracy (%)	(*p*-Value)
Unequipped	87.51	87.01	(1.000)	**73.16**	(<0.001) *	86.82	(1.000)	86.22	(1.000)
Equipped	84.78	83.97	(0.272)	**68.61**	(<0.001) *	83.97	(0.787)	84.28	(1.000)

**Table 8:** Mean percentage of correctly classified instances for each classification algorithm. (**a**) Model trained and tested with unequipped state data; (**b**) model trained with unequipped state data, tested with equipped state data; (**c**) model trained and tested with equipped state data.

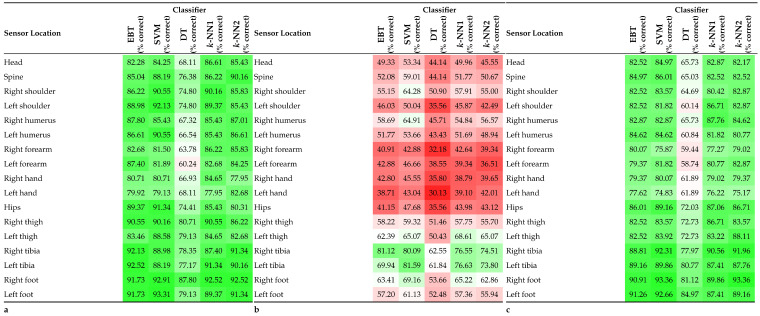

**Table 9:** Classifier (SVM) accuracy for each number of sensor combinations.

				95% Confidence Int.			
Sensor Combination	N	Mean (%)	Std.Dev (%)	Lower Bound (%)	Upper Bound (%)	Min (%)	Max (%)
One sensor	17	84.78	5.39	82.01	87.55	74.83	93.36
Two sensors	136	93.27	1.96	92.94	93.61	85.80	96.29
Three sensors	680	95.37	1.22	95.28	95.46	89.09	97.76
Four sensors	2380	96.37	0.81	96.34	96.40	91.19	98.11
Five sensors	6188	96.93	0.62	96.91	96.94	93.43	98.39
Six sensors	12,376	96.36	1.20	96.34	96.38	89.92	99.99
Seven sensors	19,448	96.66	1.12	96.65	96.68	90.48	99.99
Eight sensors	24,310	96.88	1.06	96.87	96.90	90.76	99.99
Nine sensors	24,310	97.05	1.01	97.04	97.06	91.88	99.72
Ten sensors	19,448	97.20	0.96	97.18	97.21	91.88	99.99
Eleven sensors	12,376	97.29	0.96	97.27	97.30	92.16	99.99
Twelve sensors	6188	97.39	0.90	97.37	97.42	92.72	99.99
Thirteen sensors	2380	97.49	0.88	97.45	97.52	93.00	99.72
Fourteen sensors	680	97.61	0.85	97.55	97.68	94.40	99.44
Fifteen sensors	136	97.62	0.79	97.49	97.75	95.24	99.16
Sixteen sensors	17	97.51	0.96	97.02	98.01	94.96	98.88
Seventeen sensors	1	98.60	0.00	98.60	98.60	98.60	98.60

**Table 10:** One to ten (and worst) ranked sensors for SVM classifier (equipped state); (**a**) single sensor, (**b**) two-sensor combination.

(a)		
**Rank**	**Sensors**	**Accuracy (%)**
1	Right foot	93.36
2	Left foot	92.66
3	Right tibia	92.31
4	Left tibia	89.86
5	Hips	89.16
6	Spine	86.10
7	Head	84.97
8	Left humerus	84.62
9	Left thigh	83.92
10	Right shoulder	83.75
17	Left hand	74.83
(**b**)		
1	Hips and right tibia	96.29
2	Right humerus and left foot	95.87
3	Right shoulder and right foot	95.87
5	Spine and right foot	95.73
5	Hips and right foot	95.73
6	Left thigh and left foot	95.73
7	Right humerus and right tibia	95.66
8	Right humerus and right foot	95.59
9	Right tibia and left foot	95.52
10	Head and hips	95.45
136	Left forearm and left hand	85.80

## Data Availability

The data presented in this study are available on request from the corresponding author.
